# Evaluation of the Incidence of Pharyngocutaneous Fistula after Total Laryngectomy

**DOI:** 10.22038/IJORL.2023.69853.3370

**Published:** 2023-05

**Authors:** Maziar Motiee Langaroudi, Mehrdad Jafari, Roxana Safari, Mehraveh Sadeghi Ivraghi, Alireza Mazarei

**Affiliations:** 1 *Department of Otorhinolaryngology, Tehran University of Medical Sciences, Tehran, Iran.*; 2 *Department of Medicine, School of Medicine, Tehran University of Medical Sciences, Tehran, Iran.*; 3 *Department of Medicine, School of Medicine, Qazvin University of Medical Sciences, Qazvin, Iran. *; 4 *Otorhinolaryngology Research Center, Tehran University of Medical Sciences, Tehran, Iran.*

**Keywords:** Pharyngocutaneous Fistula, Laryngectomy, Incidence, Risk Factors

## Abstract

**Introduction:**

Laryngeal squamous cell carcinoma is one of the most critical head and neck cancers. Total laryngectomy is one of the main options for treating laryngeal squamous cell carcinoma responsible for forming pharyngocutaneous fistula (PCF), which increases morbidity and mortality. This study aimed to determine PCF incidence and identify the factors associated with this complication.

**Materials and Methods:**

In a retrospective cohort study, 85 patients who underwent total laryngectomy at Imam Khomeini Hospital (Tehran, Iran) from 2011 to 2019 were selected as the study population. The presence/absence of PCF, weight, anemia status (Hb <12.5 g/dl), renal dysfunction status (GFR <90 mL/min/1.73m2), malnutrition status (Albumin <3.5 g/dl), and marginal involvement status was extracted from postoperative medical records. The data were analyzed using SPSS ver. 26.0.

**Results:**

The overall incidence of PCF was 11.8%. The mean ±SD of the duration of hospitalization in patients with PCF was 32.40 ±14.75 days, and in patients without PCF was 16.89 ±7.05 days (P = 0.009). The mean ±SD of time to develop a fistula was 7.4 ±3.74 days.

**Conclusions:**

The statuses of anemia, malnutrition, renal dysfunction, surgical margin, history of radiotherapy, pharynx closure, gender, and age were unrelated to the incidence of PCF. Further studies with a larger sample size are recommended.

## Introduction

Laryngeal squamous cell carcinoma is one of the most critical head and neck cancers. As one of the main treatments for laryngeal cancer, total laryngectomy can lead to complications, such as wound infection, dysphagia, airway obstruction, chyle leak, and carotid artery rupture (1). One of the most critical complications of total laryngectomy is pharyngocutaneous fistula (PCF) (2). 

PCF is connecting pathway between the skin and pharynx that causes saliva and nutrients to reach the skin. Morbidities in PCFs are due to increased hospitalization time, delayed oral feeding, and risks of reoperation. Mortality increases due to delayed adjuvant radiotherapy and increased risk of severe vascular bleeding (3). It is often self-limiting, and patients receive only supportive care. However, this complication can sometimes cause extensive damage that requires reoperation (4). The overall incidence of PCF after total laryngectomy varies between 20% - 34% in different studies (1,5-7).

Bril et al. (2019) showed that PCFs were associated with preoperative skeletal muscle mass (8). According to Bulğurcu et al. (2018), oral feeding initiation after total laryngectomy is unrelated to PCFs formation (9). According to previous studies, various factors such as the history of tracheostomy (1,5,6), preoperative anemia, heavy smoking, history of radiotherapy, diabetes, and poor nutrition before surgery, postoperative Hb and surgical techniques are associated with PCF formation (1,5-7). This study aimed to evaluate PCF incidence after total laryngectomy and its related factors.

## Materials and Methods

This retrospective cohort study examined the records of patients who underwent total laryngectomy at Imam Khomeini Hospital from 2011 to 2019. After excluding patients with incomplete information, 85 were selected as the study population. The presence or absence of a pharyngocutaneous fistula for each patient was determined according to the course of the disease in the hospital record. Patients' weights during surgery were also extracted to calculate the G Glomerular filtration rate (GFR). For variables such as anemia, malnutrition, and renal dysfunction, According to the results of blood tests, hemoglobin (Hb), Albumin, and creatinine (Cr) of patients were extracted during hospitalization and before surgery. Anemia was characterized by Hb <12.5 g/dl, malnutrition by Albumin <3.5 g/dl, and renal dysfunction by GFR <90 mL/min/1.73m2. GFR was calculated using patients' Cr, age, weight, and sex according to the Cockcroft-Gault formula. Marginal involvement was extracted from postoperative pathology results. Finally, the data were analyzed using Statistical Package for the Social Sciences (SPSS) version 26.0 software (IBM Corp). The overall incidence of PCF and the relationship between PCF and the study variables were examined and analyzed.

Imam Khomeini Hospital Complex-Tehran University of Medical Sciences Ethics Committee (IR.TUMS.IKHC.REC.1398.080). 

## Results

In the present study, 85 patients who underwent total laryngectomy were studied. The overall incidence of PCF was 11.8% (n =10). Our results showed that the mean ±SD of time for fistula development was 7.4 ±3.74 days.

Also, the mean ±SD of the age of patients with PCF was 61 ± 12.26-year-old, while the mean ±SD of the age of patients without PCF was 60.65 ± 9.95-year-old; there was no statistically significant difference (P=0.933). The incidence of PCFs between those who had previous radiotherapy and those who had not was 18.2% and 7.7%, respectively, but this difference was not statistically significant (P =0.176). 

Sixty-six patients had primary closure, and 19 patients had closure with flap. The incidence of PCF was higher in patients with flap closure (15.8%) than in patients with primary closure (10.6%); however, this difference was not statistically significant (P =0.686). PCF was more common in patients with anemia (15.2%) than in those with normal hemoglobin (7.7%). However, this difference was not statistically significant (P=0.331). 

Similarly, the prevalence of PCF was insignificantly higher in patients with malnutrition (20%) than in patients without malnutrition (9.2%, P = 0.236). PCF was calculated to be 15.4% (n =8) in patients with renal dysfunction and 6.1% (n =2) in patients with normal renal function; however, the difference was insignificant (P= 0.303). The positive surgical margin did not make a significant difference in the incidence of PCF (P =0.667). However, patients with PCF were significantly longer hospitalization duration than others (P=0.009). The mean ±SD hospitalization duration was 32.40 ± 14.75 days in patients with PCF and 16.89 ± 7.05 days in others (Table 1).

**Table 1:** Patient characteristics and univariate analysis

**Variable**		**Fistula status**		**Chi-Square**	**P-value**
		**With fistula** **N (%)**	**Without fistula** **N (%)**		
Patients count (n)		10 (11.8%)	75 (88.2%)	-	-
Age (years)		61.01 ± 12.26*	60.65 ± 9.95*	0.086	0.933
Hospitalization duration (days)		32.4 ± 14.75*	16.89 ± 7.051*	-3.275	**0.009**
Fistula formation time (days)		7.4 ± 3.74*	-	-	-
Gender	Male	10 (12.2%)	72 (87.8%)	0.415	1.000
	Female	0 (0%)	3 (100%)		
Previous radiotherapy	Yes	6 (18.2%)	27 (81.8%)	2.140	0.176
	No	4 (7.7%)	48 (92.3%)		
Pharynx closure	Primary	7 (10.6%)	59 (89.4%)	0.382	0.686
	Flap	3 (15.8%)	16 (84.2%)		
anemia	Yes	7 (15.2%)	39 (84.8%)	1.151	0.331
	No	3 (7.7%)	36 (92.3%)		
Malnutrition	Yes	4 (20%)	16 (80%)	1.709	0.236
	No	6 (9.2%)	59 (90.8%)		
Renal dysfunction	Yes	8 (15.4%)	44 (84.6%)	1.691	0.303
	No	2 (6.1%)	31 (93.9%)		
Positive margins	Yes	2 (14.3%)	12 (85.7%)	0.103	0.667
	No	8 (11.3%)	63 (88.7%)		
					

## Discussion

Total laryngectomy is one of the main options for treating laryngeal squamous cell carcinoma, which has not been replaced by advanced treatment approaches and radiotherapy techniques for resistant and recurrent cases (10,11). PCF is one of the most critical complications of total laryngectomy, which delays oral feeding, patient discharge, and adjuvant radiotherapy and increases the risk of reoperation, severe vascular hemorrhage, mortality, and morbidity (2,3).

The lowest and highest incidence of PCFs reported in recent studies were 5.1% and 49.6%, respectively (2,12). In our study, the incidence of PCF was 11.8%, which is a low rate compared to similar studies. In the present study, the mean length of hospitalization duration in patients with and without PCF was 32.4 days and 16.89 days, respectively, which was statistically significant (P =0.009). Busoni et al. reported an average hospitalization duration of 51.6 days in patients with fistula and 19.8 days in patients without fistula, which was higher than our findings. 

However, similar to our results, it was shown that PCF could significantly increase hospitalization duration (P =0.003) (4). Also, in the study of Koob et al., the mean hospitalization duration of patients with and without PCF was 59 days and 26 days, respectively (P <0.001) (13). Therefore, PCF is likely to be associated with increased hospitalization duration.

In the present study, fistula formation after total laryngectomy took an average of 7.4 days. Saki et al. reported an average time of 9.6 days for postoperative fistula formation, which is close to our results (14). 

Also, Aslier et al. reported this amount as 8.07 days (6). In a study by Dedivitis et al., PCF development occurred between 3 and 8 days after surgery (15). The fistula was diagnosed between 4 and 16 days after surgery, covering a more comprehensive range in our research.

Risk factors for PCF have been evaluated in various studies. Association of PCF with variables such as age, sex, BMI, smoking, tumor grading, stage, diabetes, hypertension, anemia, preoperative hemoglobin, preoperative Albumin, closure type, previous tracheostomy, previous chemotherapy, history of radiotherapy, Neck dissection, surgical margin, Etc. have been evaluated in previous studies(2,3,6,7). However, the results were controversial. Therefore, this study examined the relationship between PCF formation and variables such as age, sex, radiotherapy history, surgical margin, closure type, preoperative hemoglobin, preoperative Albumin, and renal dysfunction. 

In our study, the mean age of patients with and without PCF was 61 and 60.65 years, respectively, which was not statistically significant (P =0.933). Saki et al. reported a similar mean age in patients with and without PCF (54.2 vs. 55.3 years) (14). Aires et al. showed that PCF incidence was higher in patients older than 60, but this difference was insignificant (P =0.81) (12). In contrast, Nitassi et al. reported a considerable difference between the mean age of patients with and without PCF (61.29 and 56.68 years, respectively), which suggests aging is a risk factor for PCFs (5). In the study of Dedivitis et al., PCFs were significantly associated with age over 60 years (P =0.05) (15).

The current study showed no significant relationship between gender and PCF development. 12.2% of men and 0% of women presented this complication (P =1.00). Aires et al. showed a higher incidence of PCF in females without a significant sex relationship (P =0.18) (12). Similar to our findings, Vasani et al. reported a higher incidence of PCF in males but showed no significant relationship between fistula incidence and sex (P=0.59) (3). According to Aslier et al., the incidence of fistula in women (40%) was higher than in men (19%), but the sex difference was not significant (P =0.119) (6). 

In the present study, PCFs were more common in patients with a history of preoperative radiotherapy, but no significant association was observed between PCF formation and previous radiotherapy. Similar results were reported by Mattioli et al. (7). 

However, Dedivitis et al. showed a slightly higher incidence of PCF in patients with no history of radiotherapy (P =0.354) (15). In contrast, in the studies of Saki et al. and Aslier et al., the history of radiotherapy had a significant positive association with PCF (6, 14). This contrast can be explained by a decline in tissue repair capacity after radiotherapy (14).

In the present study, PCF was more common in patients with flap closure (15.8%) than in patients with primary closure (10.6%), and this association was statistically insignificant (P =0.686). Similar results were reported by Nitassi et al. (5). Also, Saki et al. mentioned that PCFs were unrelated to closure type and surgical technique (14).

 In contrast, Benson et al. showed that the Pectoralis flap incorporated into the suture line was associated with PCF (P =0.02). Patients with primary closure had the lowest incidence of PCF (1). Also, Lebo et al. reported free flap reconstruction as a risk factor for PCF compared with primary closure (2). Due to the low frequency of closure with flap and incidence of PCF in our study, the lack of significant association is reasonable. It is best to conduct future studies with larger sample sizes.  In patients with anemia, the incidence of PCF was 15.2%, and in non-anemic patients was 7.7%, which was not statistically significant (P =0.331). Similarly, Busoni et al. considered preoperative Hb <12.5 g/dl as anemia and did not report a significant association with PCF (P =0.073) (4). Mattioli et al. defined anemic patients as Hb <12.2 g/dl before surgery and indicated no association between anemia and PCF (P =0.14) (7). Nitassi et al. considered Hb <12 g/dl as the definition of anemia and reported a significant association between anemia and PCF (P =0.035) (5). The lower hemoglobin cutoff can explain this conflict in Nitasi et al. than in our research and other studies.In the present study, the incidence of PCF was insignificantly higher in patients with malnutrition than in patients without malnutrition (P =0.236). 

Busoni et al. and Cecatto et al. failed to demonstrate a significant association between malnutrition and PCF formation (4,16). While in the study of Mattioli et al., preoperative Albumin <3.5 was significantly associated with PCF formation (P =0.005) (7).

In this study, we considered GFR <90 ml/min/1.73/m2 as renal dysfunction. Due to the high mean age of the patients (60.69 years) and the annual reduction of 0.75 ml/min in GFR, renal dysfunction was observed in 52 patients, which was relatively high (17).

The incidence of PCF in patients with renal impairment (15.4%) was higher than in patients with normal renal function (6.1%), which was not statistically significant. (P =0.303). No research investigated the relationship between renal function and PCF incidence. In the study of Benson et al., comorbidity was introduced as one of the risk factors for PCF (P =0.04) (1). 

Saki et al. identified systemic disease as a risk factor for PCF (14).In our study, the incidence of PCF was 14.3% in patients with a positive surgical margin and 11.3% in patients with a negative surgical margin, with no significant difference (P =0.667). Similarly, Benson et al. and Vasani et al. did not demonstrate a significant association between surgical margin and PCF (P =0.999) (1,3). In contrast, Saki et al. found PCFs associated with a positive surgical margin (14).

## Conclusion

Pharyngocutaneous fistula (PCF) is a common complication after total laryngectomy, which leads to increased hospitalization time and morbidity. In our study, PCFs were not associated with age, anemia, malnutrition, renal impairment, surgical margin, history of radiotherapy, closure, and gender. However, we suggest that more studies with higher sample sizes be performed to investigate the association of this complication with such factors.

## References

[1] Benson EM, Hirata RM, Thompson CB, Ha PK, Fakhry C, Saunders JR (2015). Pharyngocutaneous fistula after total laryngectomy: a single-institution experience, 2001–2012. American journal of otolaryngology..

[2] Lebo NL, Caulley L, Alsaffar H, Corsten MJ, Johnson-Obaseki S (2017). Peri-operative factors predisposing to pharyngocutaneous fistula after total laryngectomy: analysis of a large multi-institutional patient cohort. Journal of Otolaryngology-Head & Neck Surgery..

[3] Vasani SS, Youssef D, Lin C, Wellham A, Hodge R (2018). Defining the low‐risk salvage laryngectomy—A single‐center retrospective analysis of pharyngocutaneous fistula. Laryngoscope investigative otolaryngology..

[4] Busoni M, Deganello A, Gallo O (2015). Pharyngocutaneous fistula following total laryngectomy: analysis of risk factors, prognosis and treatment modalities. Acta Otorhinolaryngologica Italica..

[5] Nitassi S, Belayachi J, Chihab M, Rkain I, Benayad J, Benbouzid MA (2016). Evaluation of post laryngectomy pharyngocutaneous fistula risk factors. Iranian journal of otorhinolaryngology..

[6] Aslıer NGY, Doğan E, Aslıer M, İkiz AÖ (2016). Pharyngocutaneous fistula after total laryngectomy: risk factors with emphasis on previous radiotherapy and heavy smoking. Turkish archives of otorhinolaryngology..

[7] Mattioli F, Bettini M, Molteni G, Piccinini A, Valoriani F, Gabriele S (2015). analysis of risk factors for pharyngocutaneous fistula after total laryngectomy with particular focus on nutritional status. Acta Otorhinolaryngologica Italica..

[8] Bril SI, Pezier TF, Tijink BM, Janssen LM, Braunius WW, de Bree R (2019). Preoperative low skeletal muscle mass as a risk factor for pharyngocutaneous fistula and decreased overall survival in patients undergoing total laryngectomy. Head & neck..

[9] Bulğurcu S, Çukurova İ (2018). Comparison of early versus delayed oral feeding after total laryngectomy in terms of pharyngocutaneous fistula development. Turkish archives of otorhinolaryngology..

[10] Fasunla AJ, Ogundoyin OA, Onakoya PA, Nwaorgu OG (2016). Malignant tumors of the larynx: Clinicopathologic profile and implication for late disease presentation. Nigerian medical journal: journal of the Nigeria Medical Association..

[11] Birkeland AC, Beesley L, Bellile E, Rosko AJ, Hoesli R, Chinn SB (2017). Predictors of survival after total laryngectomy for recurrent/persistent laryngeal squamous cell carcinoma. Head & neck..

[12] Aires F, Dedivitis R, Kulcsar M, Ramos D, Cernea C (2018). Neutrophil-to-lymphocyte ratio as a prognostic factor for pharyngocutaneous fistula after total laryngectomy. Acta Otorhinolaryngologica Italica..

[13] Koob I, Pickhard A, Buchberger M, Boxberg M, Reiter R, Piontek G (2020). Bradykinin Receptor B1 and C-Reactive Protein as Prognostic Factors for Pharyngocutaneous Fistula Development After Laryngectomy. Head and neck pathology..

[14] Saki N, Nikakhlagh S, Kazemi M (2008). Pharyngocutaneous fistula after laryngectomy: incidence, predisposing factors, and outcome. Arch Iran Med..

[15] Dedivitis R, Ribeiro K, Castro M, Nascimento P (2007). Pharyngocutaneous fistula following total laryngectomy. Acta otorhinolaryngologica italica..

[16] Cecatto SB, Monteiro-Soares M, Henriques T, Monteiro E, Moura CIFP (2015). Derivation of a clinical decision rule for predictive factors for the development of pharyngocutaneous fistula postlaryngectomy. Brazilian journal of otorhinolaryngology..

[17] Hommos MS, Glassock RJ, Rule AD (2017). Structural and functional changes in human kidneys with healthy aging. Journal of the American Society of Nephrology..

